# Characteristics and Antitumor Activity of Doxorubicin-Loaded Multifunctional Iron Oxide Nanoparticles in MEC1 and RM1 Cell Lines

**DOI:** 10.3390/jfb15120364

**Published:** 2024-12-03

**Authors:** Nino Maisuradze, Shalva Kekutia, Jano Markhulia, Tamar Tsertsvadze, Vladimer Mikelashvili, Liana Saneblidze, Nikoloz Chkhaidze, Zsolt Endre Horváth, László Almásy, Nunu Mitskevichi

**Affiliations:** 1 Division of Immunology and Microbiology, Iv. Javakhishvili Tbilisi State University, 1, Ilia Tchavchavadze Ave., 0179 Tbilisi, Georgia; 2 Nanocomposites Laboratory, Vladimer Chavchanidze Institute of Cybernetics of the Georgian Technical University, Z. Anjafaridze Str. 5, 0186 Tbilisi, Georgia; 3 Institute for Technical Physics and Materials Science, HUN-REN Centre for Energy Research, Konkoly-Thege Miklós Str. 29-33, 1121 Budapest, Hungary; 4 Institute for Energy Security and Environmental Safety, HUN-REN Centre for Energy Research, Konkoly-Thege Miklós Str. 29-33, 1121 Budapest, Hungary

**Keywords:** nanoparticles, cancer therapy, doxorubicin, drug delivery, cell cultures

## Abstract

The rapid progress in nanotechnology has introduced multifunctional iron oxide nanoparticles as promising agents in cancer treatment. This research focused on the synthesis and assessment of citric-acid-coated, folic-acid-conjugated nanoparticles loaded with doxorubicin, evaluating their therapeutic potential in tumor models. An advanced automated continuous technology line (CTL) utilizing a controlled co-precipitation method was employed to produce highly dispersive, multifunctional nanofluids with a narrow size distribution. Various techniques, including dynamic light scattering (DLS), electrophoretic light scattering (ELS), X-ray diffraction (XRD), and transmission electron microscopy (TEM), were employed to examine the particle size, zeta potential, structure, and morphology. Magnetic properties were analyzed through vibrating sample magnetometry (VSM), and surface modifications were confirmed via UV-visible (UV-Vis) and Fourier-Transform Infrared (FTIR) spectroscopy. Cytotoxicity and drug delivery efficiency were evaluated in vitro using RM1 (prostate cancer) and MEC1 (chronic lymphocytic leukemia) cell lines. Fluorescence microscopy demonstrated the successful intracellular delivery of doxorubicin, showcasing the nanoparticles’ potential for targeted cancer therapy. However, folic-acid-conjugated nanoparticles exhibited diminished effectiveness over time. This study highlights the importance of nanoparticle optimization for enhancing therapeutic performance. Further research should aim to improve nanoparticle formulations and explore their long-term impacts for the development of safe, targeted cancer treatments.

## 1. Introduction

In recent years, researchers have shown that the prospect of using magnetically controlled nanoparticles in medicine is becoming more and more actual [1]. The nanocomposites created on their basis are equipped with the functions of medical nanobots [2], such as the ability to distinguish microbiological objects in the biological area [3], the targeted transportation of medicinal drugs to the target organ [4,5], the diagnosis and therapy of diseases at the cellular level [6], and the adsorption and removal of toxins through a magnetic field [7]. The results of physical, chemical, and biological experiments confirm the potential of multifunctional magnetic nanoparticles as medicinal drug delivery systems [1,3]. However, at the same time, various aspects of the interaction of magnetic nanoparticles with living organisms and cells are still relevant [8]. The consequences of the damaging effects of nanomaterials on the cellular structure, biodegradation processes of nanoparticles in the biological medium, dependence of toxicity on the nanosystem concentration, etc., have not been fully studied [9].

The main goal of this research was the synthesis of medical multifunctional iron oxide nanoparticles and the study of their effectiveness on an experimental tumor model. Continuing our previous research [10,11], here, we evaluate the action of different configurations of magnetic nanoparticles loaded with an antitumor chemotherapy agent, doxorubicin (DOX) [12], in an in vitro tumor model, in order to obtain a new prototype of an effective antitumor therapeutic nanosystem with fewer side effects. To achieve this goal, it was necessary to create an optimal tumor model in which preliminary in vitro studies were conducted on different tumor cell lines. As a result, it was possible to evaluate the antitumor efficiency of different concentrations of DOX-loaded nanoparticles and to select the least resistant culture for further studies.

Recent studies have shown that magnetically controlled nanoparticles hold significant promise in the treatment of neurodegenerative diseases such as Alzheimer’s and Parkinson’s [13]. For instance, nanoparticles functionalized with specific ligands can cross the blood–brain barrier and target amyloid plaques, a hallmark of Alzheimer’s disease [14]; these nanoparticles can be engineered to deliver therapeutic agents directly to the brain, potentially reducing the progression of neurodegenerative symptoms [15]. In Parkinson’s disease, it was demonstrated that magnetic nanoparticles could facilitate the delivery of neuroprotective drugs to dopaminergic neurons, offering a targeted approach to mitigate neuronal loss and improve motor function [16].

Moreover, infectious diseases are another critical area where magnetically controlled nanoparticles are making an impact. Recent advancements have explored the use of these nanoparticles in the targeted treatment of bacterial infections, particularly those caused by antibiotic-resistant strains [17]. For example, one of the recent studies developed magnetically guided nanoparticles that can deliver antibiotics directly to the site of infection, enhancing the drug’s efficacy while minimizing systemic side effects [18]. In addition, some researchers have shown that these nanoparticles can be designed to disrupt bacterial biofilms, which are often resistant to conventional treatments, thereby providing a potent tool against persistent infections [19].

Prostate cancer is another area where magnetically controlled nanoparticles have shown substantial potential. Prostate cancer remains one of the most common cancers among men, and traditional treatments such as surgery, radiation, and chemotherapy often come with significant side effects [20]. One of the promising recent research projects has demonstrated that iron oxide nanoparticles can be used to specifically deliver chemotherapeutic agents to prostate cancer cells, reducing the impact on surrounding healthy tissues [21]. These nanoparticles can be magnetically guided to the tumor site, ensuring that a higher concentration of the drug is delivered directly to the cancer cells, thereby improving treatment efficacy and minimizing systemic toxicity.

Chronic lymphocytic leukemia (CLL), a type of cancer that affects the blood and bone marrow, has also been a focus of nanoparticle research. Traditional treatments for CLL, such as immunotherapy and chemotherapy, can cause severe side effects and may not always be effective in advanced stages [22]. Recent studies have explored the use of magnetic nanoparticles to deliver targeted therapies directly to leukemic cells [23]. These nanoparticles can be engineered to carry therapeutic agents that specifically bind to cancerous B-cells, which are characteristic of CLL. This targeted approach not only enhances the effectiveness of the treatment but also significantly reduces adverse effects, offering new hope for patients with CLL.

By integrating these recent findings, we can appreciate the broad potential of magnetically controlled nanoparticles in addressing some of the most challenging medical conditions of our time. Their ability to deliver targeted therapy with precision not only enhances treatment efficacy but also reduces side effects, paving the way for more effective and safer medical interventions.

Magnetically controlled nanoparticles are emerging as a promising tool in medicine, offering targeted drug delivery, diagnosis, and even toxin removal. Recent research has highlighted their potential in treating various diseases, including cancer [24]. One of our recent findings demonstrated the effectiveness of doxorubicin (DOX)-loaded citric-acid-modified magnetic nanoparticles in inhibiting the growth and proliferation of triple-negative breast cancer cells [11]. This study highlights the potential of these nanoparticles for targeted drug delivery, minimizing side effects, and enhancing the cytotoxic effect of DOX on cancer cells.

This research aimed to investigate the potential of magnetic nanoparticles in cancer therapy by synthesizing and evaluating the effectiveness of various nanoparticle configurations. Initially, we tested these configurations on RM1 cell lines, where previous studies had shown promising results [10]. Following this, we examined their effects on treatment-resistant MEC1 in vitro cell lines [25]. Our goal was to develop a novel prototype of an effective antitumor therapeutic nanosystem that minimizes side effects while also serving as a successful drug delivery carrier.

## 2. Materials and Methods

**Synthesis of Nanoparticles.** The synthesis of magnetic nanofluid containing citric-acid-coated (or folium-acid-conjugated) and DOX-loaded iron oxide nanoparticles was carried out by modifying the standard chemical co-precipitation procedure in an automated continuous technology line [26]. The dissolution processes were carried out in a reactor under reduced pressure in an inert gas (N_2_) atmosphere using a Schlenk line connected to an automated continuous technology line. This automatization makes the synthesis process more efficient and obtains highly dispersive, multifunctionalized, reproducible nanofluids with a narrow size distribution of nanoparticles, preventing the possible unwanted oxidation of divalent iron ions (Fe^2+^). Furthermore, some samples were additionally subjected to an electrohydraulic discharge (EHD) treatment at one stage of the synthesis.

The process of nanoparticle synthesis can be divided into the following stages:

Stage I—synthesis of ferromagnetic nanofluid containing iron oxide nanoparticles: First, FeCl_3_ · 6H_2_O (9 g) (Sigma-Aldrich Co., LLC, St. Louis, MO, USA) + 333 mL of deionized water (DW) (0.1 M solution) was prepared in the jacketed reactor with mechanical stirring (temperature 45 °C, mixing duration 20 min. At the same time, 6.86 g (0.024 mol) of FeSO_4_ · 7H_2_O (Sigma-Aldrich Co., LLC, St. Louis, MO, USA) (stirring time 30 min) was dissolved in deionized water in a chemical reactor, under mechanical stirring (500 rpm) at 45 °C with an inert gas system (Schlenk line). Also, separately, 26 mL of 25% ammonium hydroxide (NH_4_OH) (Sigma-Aldrich Co., LLC, St. Louis, MO, USA) was dissolved in 66 mL of distilled water under magnetic stirring (500 rpm) at 40 °C. A separately dissolved 0.2 M iron (III) chloride aqueous solution was then added to the chemical reactor (in which 0.2 M iron (II) sulfide aqueous solution was previously prepared) via a peristaltic pump. In the vacuum medium, at 45 °C, the said iron salts were stirred with a mechanical stirrer (750 rpm) for 10 min, after which 0.2 molar ammonium hydroxide was added through a peristaltic pump (at a speed of 3 mL/min). After the complete delivery of ammonium hydroxide, the mechanical stirring (750 rpm, 45 °C) of the resulting colloidal solution in the chemical reactor was continued for 40 min.

Stage II: leaching of synthesized ferromagnetic nanofluid, ultrasonic treatment, and coating with citric acid. The magnetic colloid containing iron oxide nanoparticles was washed with distilled water by decantation on a permanent magnet to remove unwanted reaction products (ammonium chloride and sulfate), which formed as a result of the synthesis, until the pH of the suspension became equal to 6.5. Finally, the washed sample was added to 200 mL of deionized water and treated with an ultrasonic disperser at 80% power for 5 min. After that, the coating with citric acid or conjugation with folic acid was carried out by adding a pre-prepared aqueous solution of citric acid/folic acid (0.585 g CA/0.585 g FA) (Sigma-Aldrich Co., LLC, St. Louis, MO, USA) drop by drop to the colloid with a peristaltic pump, after which the ultrasonic treatment of the surfaced liquid continued for 20 min. Finally, the encapsulated ferromagnetic nanofluid was centrifuged at 4000 rpm for 10 min.

Stage III: loading of the synthesized magnetic nanoparticles with an antitumor drug (doxorubicin (Kocak Farma, Turkey)). Citric-acid/folic-acid-stabilized magnetic nanofluid (100 µg) was added to 2.5 mL of doxorubicin solution (10 mg/mL) and mixed together with the doxorubicin at room temperature under continuous shaking conditions.

In Stage IV, to eliminate the unbound doxorubicin, we utilized a neodymium magnet after the loading process. Upon the application of the magnetic field, the magnetic nanoparticles aggregated, resulting in unbound doxorubicin remaining in the supernatant. Subsequently, we measured the concentration of free doxorubicin in the supernatant using UV-visible spectrophotometry. This approach guarantees the efficient purification of the drug-loaded nanoparticles and confirms the successful extraction of unbound doxorubicin.

**Cell cultivation**. All cell lines were purchased from American Type Culture Collection (American Type Culture Collection (ATCC), Manassas, WV, USA) and cultured in RPMI-1640 supplemented with 10% fetal calf serum (FCS) and 1% Penicillin Streptomycin (Sigma-Aldrich Co., LLC, St. Louis, MO, USA). Cell culture maintenance, including multiplication, seeding, and separation, as well as freezing methods, were also employed. The cells were maintained in a controlled environment using an incubator (LEEC (Limited, Nottingham, UK). The incubator was set to maintain a CO_2_ concentration of 5% and a humidity level of approximately 95% to ensure optimal growth conditions for the cultured cells.

Cell cultures used: RM1 is a murine prostate cancer cell line that was induced in male C57BL/6 strain mice. Urogenital sinus cells of 17-day-old C57BL/6 mouse embryos were infected with Zipras/myc9 retrovirus and transplanted under the renal capsule of isogenic adult male mice. The cell line is metastatic; therefore, the tumor developed, and it was from here that the cell line was isolated. The RM1 tumor line is often used to create an experimental tumor model due to its relative resistance to certain anticancer agents [27].

The MEC1 cell line consists of transformed B lymphocytes obtained from a 58-year-old man diagnosed with stage 2 chronic lymphocytic leukemia (CLL) [28]. The cell line was established from a patient with unmutated IGHV genes who exhibited progression towards prolymphocytic leukemia. The patient was Epstein–Barr virus (EBV)-positive, consistent with most CLL patients, who also have the EBV receptor CD21 on their lymphocytes. The primary aim of establishing the MEC1 B cell line was to create an in vitro model that closely mimics CLL, facilitating the study of the biological processes involved in the disease.

**Characterization of nanoparticles**. The determination of the hydrodynamic diameters and zeta potential was conducted using dynamic light scattering (DLS) and electrophoretic light scattering (ELS) techniques with a Litesizer 500 particle analyzer from Anton Paar, Graz, Austria. The pH range studied was from 2 to 12, with a semiconductor laser as the light source (40 MW, 658 nm).

For crystal structure and phase analysis, X-ray diffraction (XRD) was employed on dried aqueous suspensions of bare SPIONs, CA-SPIONs, FA-SPIONs, DOX-FA-SPIONs, and DOX-CA-SPIONs using a DRON 3M X-ray diffractometer (PJSC ‘Bourevestnik’, St. Petersburg, Russia).

Transmission electron microscopy (TEM) was carried out on samples prepared by drop-drying suspensions on holey carbon-foil-coated copper grids using a JEOL 3010 transmission electron microscope (JEOL Ltd., Akishima, Tokyo, Japan).

Magnetization curves of magnetic nanofluids were measured with a vibrating sample magnetometer (VSM) (7300 Series VSM System, Lake Shore Cryotronics, Inc., Westerville, OH, USA) at room temperature.

FTIR spectroscopic studies were performed using a Thermo Scientific™ Nicolet™ iS™ 5 instrument (Thermo Fisher Scientific Inc., Waltham, MA, USA) with a diamond ATR crystal. UV-visible absorption measurements of doxorubicin solutions and nanoparticles were recorded on an AVAspec HS 2048XL spectrometer (Avantes, Inc., Apeldoorn, The Netherlands) in the wavelength range of 200–1100 nm at room temperature.

**Determination of optical density of cells in solutions**. The optical density (OD562) of the cell suspension was determined by analyzing filled 96-cell plates using a scanning multiscan spectrophotometer at a wavelength of 562 nm. (Optic Ivymen System, Model 2100C, Biotech SL, Madrid, Spain). The number of cells was determined at 0, 24, and 48 h. A total of 100 µL of the cell suspension was transferred to each well. After shaking the cassette three times (20 s), the absorbance at 562 nm was immediately measured. Thus, a linear relationship between OD values and cell concentrations is determined.

**Preparation of cytopreps**. The cell concentration was brought to 2 × 10^6^/mL; after fixing 100 μL of cells, the suspension was transferred to the cuvettes of a cytocentrifuge Cytospin 2 (Shandon Thermo Fisher Scientific, Waltham, MA, USA). The cuvette consisted of a slide, a metal holder, a funnel, and a paper filter. The suspension was added to the cuvette funnel. Centrifugation was performed for 10 min at 600 (RPM). Then, the samples were observed with a fluorescence microscope (Zeiss, Oberkochen, Germany). The imaging was performed using a green laser with an excitation wavelength of 532 nm, which is optimal for exciting the doxorubicin fluorescence and allowing for accurate measurements.

**MTT method**. The cytotoxicity of the samples were assessed via the MTT (3-[4,5-dimethyltetrazolium-2-yl]-2,5-diphenyltetrazolium bromide) test. This cell proliferation assay is based on the ability of the enzyme mitochondrial dehydrogenase in viable cells to disperse the bright yellow color of tetrazolium salt and convert it to a dark blue formazan crystal. The number of surviving cells is directly proportional to the number of formazan crystals, which is measured by spectrophotometry at a wavelength of 570 nm.

**Design of in vitro experiments.**

The following steps were performed:

- Cultivation of cell lines;
- Obtaining the required concentration for cell research (10,000 cells/mL);
- Making different combinations of nanoparticle-drug systems (Table 1);
- Incubation of cells and nanofluid in an environment as close as possible to the organism (37 °C, 5% CO_2_);
- Cytotoxicity assessment of samples at 0, 24, and 48 h using the MTT method;
- Experiments were conducted twice, with each sample analyzed in duplicate;
- All the experiments included a “control”—cells that were incubated without any particles added, ensuring a clear comparison to the treated samples.

The incubation of cells was carried out at 0 h, 24 h, and 48 h. The test substances were added at the specified concentrations (see Table 1), and after incubation, 10 μL of MTT solution was added and incubation was carried out for 3–4 h at 37 °C. Finally, 100 μL MTT solvent was added, gentle pipetting was performed, and the optical density was measured.

**Statistical Analysis**. The percentage of cell viability for each treated sample was calculated relative to the control group (untreated cells) using the following formula:

Cell Viability% = (Absorbance of treated sample/Absorbance of control) × 100%

Subsequently, we calculated the percentage of cytotoxicity as follows:

Cytotoxicity% = 100 − Viability%

This calculation provides a quantitative measure of the compound’s cytotoxic effect. The data presented in biological studies are the mean and their standard deviation (SD). The data were analyzed using SPSS software (version 29.02.0). The differences between treatments were determined using a two-way ANOVA. Results were considered statistically significant if the *p*-value was less than 0.05.

## 3. Results

### 3.1. Characterization of Nanoparticles

#### 3.1.1. ELS and DLS Results

**Figure 1:**
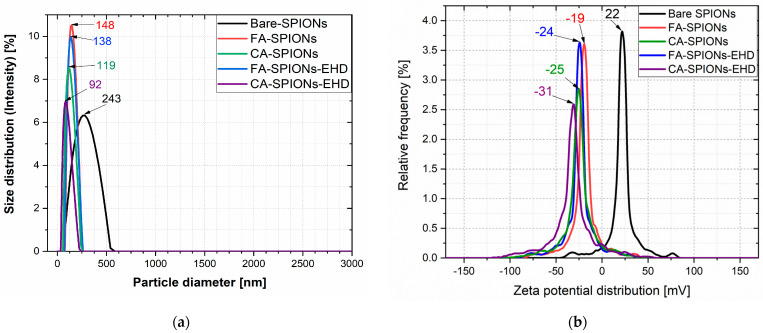
Particle size and ζ potential of Bare SPIONs; CA-SPIONs, FA-SPIONs, CA-SPIONs-EHD, and FA-SPIONs-EHD. (**a**) Particle size and (**b**) ζ potential.

The zeta potential is a measure of the electrostatic repulsion between particles in a solution. A higher absolute value of the zeta potential indicates greater stability. Both the CA-modified and FA-modified iron oxide nanoparticles and the electrohydraulic discharge-processed CA-SPIONs had negative zeta potentials from −19 mV to −31 mV, at a neutral pH (6.5) (Figure 1b). This suggests that these nanoparticles are well-dispersed in the solution and less likely to aggregate. The Bare SPIONs had a positive zeta potential of 22 mV, indicating a change in surface charge due to the CA coating or FA conjugation.

The hydrodynamic diameter refers to the size of a particle in the solution, including any surrounding hydration layer. The CA-modified iron oxide nanoparticles and the electrohydraulic discharge-processed CA-SPIONs had smaller hydrodynamic diameters of 119 nm and 92 nm, respectively, compared to FA-modified iron oxide nanoparticles and the electrohydraulic discharge-processed FA-SPIONs, with a hydrodynamic diameter of 148 nm and 138 nm, respectively (Figure 1a). However, all the modified particles were smaller than Bare-SPIONs, with a 243 nm particle diameter, likely due to the stabilization by the CA and FA molecules attached to the nanoparticle surface.

#### 3.1.2. XRD Results

**Figure 2:**
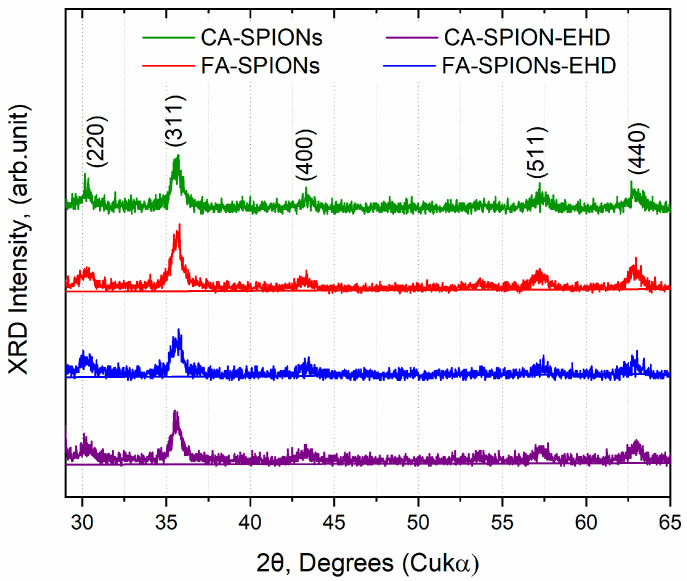
Diffraction patterns of the synthesized samples: CA-SPIONs, FA-SPIONs, CA-SPIONs-EHD, and FA-SPIONs-EHD.

X-ray diffraction (XRD) is a technique used to identify the crystal structure of a material. The XRD patterns of all synthesized samples (CA-SPIONs, FA-SPIONs, CA-SPIONs-EHD, and FA-SPIONs-EHD) showed the same characteristic peaks corresponding to the cubic inverse spinel structure of magnetite (Fe_3_O_4_) (Figure 2). This indicates that the modification process with citric acid and the conjugation with folic acid did not alter the fundamental crystal structure of the iron oxide nanoparticles.

#### 3.1.3. TEM Results

**Figure 3:**
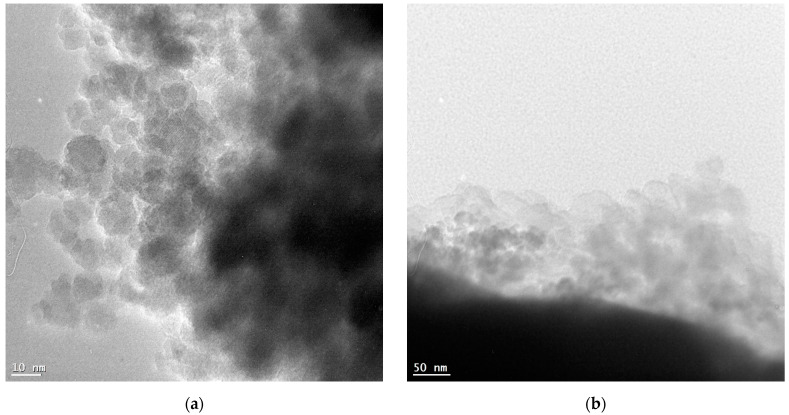
TEM images of (**a**) CA-modified and (**b**) FA-modified iron oxide nanoparticles.

Transmission electron microscopy (TEM) was used to visualize the morphology and size of nanoparticles. The TEM images revealed that the synthesized nanoparticles had a spherical morphology with a narrow size distribution (Figure 3a,b). The CA-modified iron oxide nanoparticles (CA-SPIONs) appeared slightly sparse compared to the FA-modified nanoparticles (FA-SPIONs), which is consistent with the DLS results.

#### 3.1.4. VSM Results

**Figure 4:**
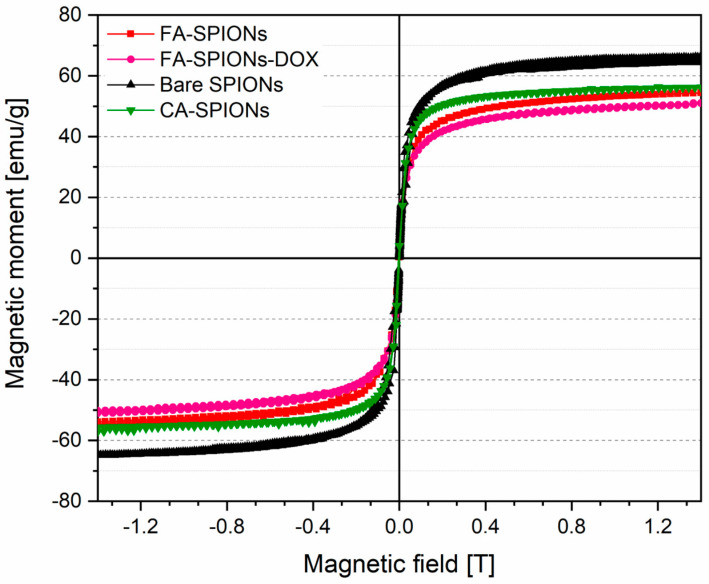
Magnetization curves of magnetic fluids with bare SPIONs, CA-coated, and FA-conjugated magnetite nanoparticles at room temperature.

Vibrating sample magnetometry (VSM) is a technique used to measure the magnetic properties of materials. The magnetization curves of the synthesized magnetic nanofluids showed that bare, folic-acid-, and citric-acid-modified iron oxide nanoparticles were measured at room temperature under an applied field of 1.4 Tesla (see Figure 4). The saturation magnetization (Ms) values for bare SPIONs, CA-SPIONs, FA-SPIONs, and FA-SPIONs loaded with doxorubicin (FA-SPIONs-DOX) were determined to be 65.0, 55.8, 54.1, and 50.5 emu/g, respectively. This means that they are magnetic only when an external magnetic field is applied, and they lose their magnetism when the field is removed. This property is important for potential applications in targeted drug delivery, as the nanoparticles can be guided to the tumor site using an external magnetic field. The VSM results also demonstrate a progressive decrease in saturation magnetization with each successive modification, confirming successful surface modifications and drug loading while highlighting the impact of these changes on the magnetic properties of the nanoparticles.

#### 3.1.5. UV-Vis Optical Studies

**Figure 5:**
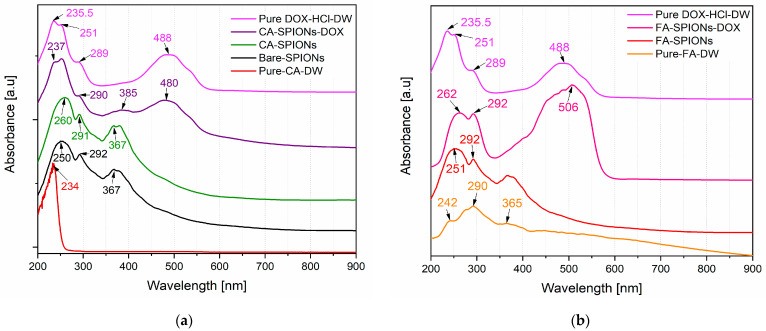
UV-VIS spectra of magnetic nanoparticles containing (**a**) Bare SPIONs; CA-SPIONs-DOX; pure DOX; CA-SPIONs; and pure CA; (**b**) FA-SPIONs-DOX; FA-SPIONs; pure CA; and pure DOX.

UV-Vis spectroscopy is a technique used to identify and quantify the presence of specific molecules based on their absorption of light at different wavelengths. The UV-Vis absorption spectra of the DOX-loaded samples showed a characteristic absorption peak at around 480 nm, which is the absorption peak of doxorubicin. The absorption spectra for pure components and nanoparticle variants (bare SPIONs, CA-SPIONs, FA-SPIONs, and drug-loaded CA-SPIONs and FA-SPIONs) were analyzed. Doxorubicin’s main absorption peaks appeared at 235, 251, 289, and 488 nm (Figure 5a,b), consistent with reported data [29,30].

The intensity of this peak increased with the increasing doxorubicin loading concentration, confirming the successful loading of doxorubicin onto the CA-SPIONs; peaks were observed at 237, 290, and 480 nm, confirming doxorubicin loading, with slight shifts indicating interaction with the nanoparticles. Citric acid exhibited a peak at 234 nm, and bare SPIONs showed characteristic peaks at 252, 292, and 367 nm (Figure 5a). After citric acid coating, slight shifts were observed, confirming a successful surface modification (Figure 5a). The spectrum of FA-SPIONs-DOX also exhibited similar features (Figure 5b), further confirming successful drug loading. This is important for the development of a drug delivery system, as it demonstrates that the nanoparticles can effectively encapsulate and carry the anticancer drug.

#### 3.1.6. FTIR Analysis

**Figure 6:**
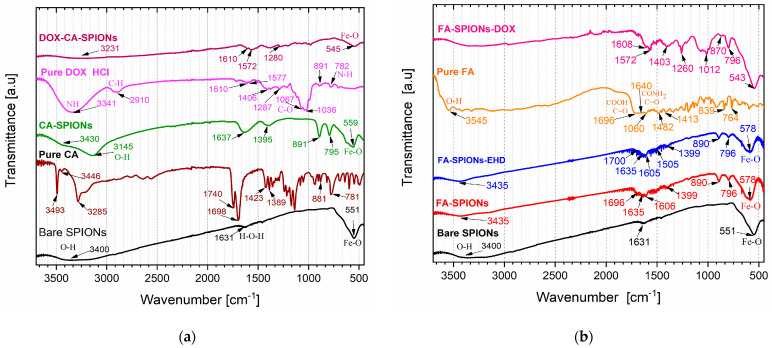
FTIR spectra of (**a**) bare SPIONs; pure citric acid (CA); CA-SPIONs; pure DOX; and DOX-CA-SPION. (**b**) bare SPIONs; pure folic acid (FA); DOX-FA-SPIONs; FA-SPIONs; and FA-SPIONs-EHD.

The FTIR analysis is crucial for confirming the successful synthesis and characterization of the nanoparticles. It provides evidence for the presence of the desired coating, such as citric acid (CA), or conjugation, such as folic acid (FA), on the iron oxide nanoparticles. Bare SPIONs exhibited peaks at 3400 cm^−1^ (O-H stretching), 1631 cm^−1^ (H-O-H bending), and 551 cm^−1^ (Fe-O stretching), confirming the presence of surface hydroxyl groups and the iron oxide core (Figure 6a). CA-SPIONs showed shifts to 3430 cm^−1^ and 1637 cm^−1^, indicating citric acid attachment. FA-SPIONs displayed characteristic peaks of folic acid, including 3435 cm^−1^ (N-H stretching) and 1631 cm^−1^ (amide I band), with the iron oxide peak remaining intact, confirming successful conjugation (Figure 6a). The distinct peaks observed in the spectra of CA-SPIONS and FA-SPIONS, compared to the bare SPIONs, confirm the successful attachment of these coatings.

Additionally, the presence of doxorubicin peaks in the DOX-CA-SPIONS spectrum peaks at 3231 cm^−1^ and 1610 cm^−1^ confirm drug loading (Figure 6a). FA-SPIONs-DOX displayed combined peaks (Figure 6b) of folic acid and doxorubicin, indicating successful drug loading onto the nanoparticles. The FTIR results provide valuable information for optimizing the synthesis process and ensuring the desired properties of the nanoparticles for their intended therapeutic applications.

### 3.2. Results of Biological Studies

#### 3.2.1. Effects of Nanoparticles on RM1 Cell Lines

**Figure 7:**
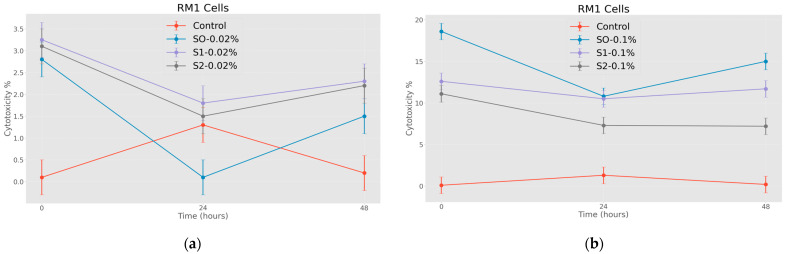
Effects of nanoparticles on RM1 cell line (MTT assay—cytotoxicity assessed at 570 nm), where control represents untreated tumor cells; (**a**) S0-0.02%—cells treated with 0.20 mg/mL concentrations of superparamagnetic iron oxide nanoparticles; S1-0.02%—cells treated with 0.20 mg/mL concentration of superparamagnetic iron oxide nanoparticles coated with citric acid; S2-0.02%—cells treated with 0.20 mg/mL concentration of electrohydraulic-processed superparamagnetic iron oxide nanoparticles coated with citric acid. (**b**) S0-0.1%—cells treated with 1.00 mg/mL concentrations of superparamagnetic iron oxide nanoparticles; S1-0.1%—cells treated with 1.00 mg/mL concentration of superparamagnetic iron oxide nanoparticles coated with citric acid; S2-0.2%—cells treated with 1.00 mg/mL concentration of electrohydraulic-processed superparamagnetic iron oxide nanoparticles coated with citric acid. *p* < 0.005.

At the initial stage of the research, the cytotoxic effect of citric-acid-coated nanoparticles was studied. Based on the obtained data (Figure 7a,b), it can be concluded at a glance that the RM1 cell line is a much more stable line, and the effect of doxorubicin on them is expressed only by a small variation in the indicators of cell survival, although it is worth noting that the efficiency of both citric-acid-coated and doxorubicin-loaded nanoparticles is similar to the indicators observed in previous studies [11].

**Figure 8:**
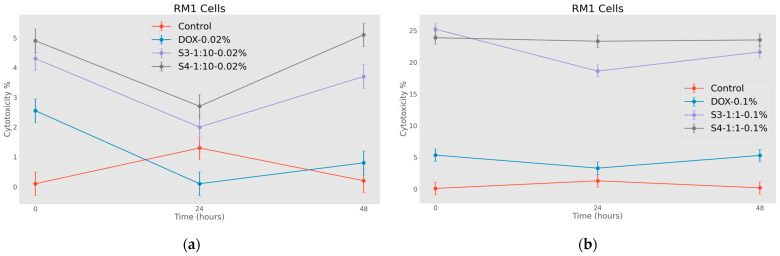
Effects of nanoparticles on RM 1 cell line (MTT assay—optical density assessed at 570 nm), where control represents untreated tumor cells; (**a**) DOX-0.02%—cells treated with doxorubicin at 0.37 mM concentrations; S3-1:10-0.02%—cells treated with 0.18 mg/mL concentrations of superparamagnetic iron oxide nanoparticles functionalized with doxorubicin and coated with citric acid; S4-1:10-0.02%—cells treated with 0.18 mg/mL concentrations of electrohydraulic-processed superparamagnetic iron oxide nanoparticles functionalized with doxorubicin and coated with citric acid. (**b**) DOX-0.1%—cells treated with doxorubicin at 1.84 mM, S3-1:1-0.1%—cells treated with 0.50 mg/mL concentrations of superparamagnetic iron oxide nanoparticles functionalized with doxorubicin and coated with citric acid; S4-1:1-0.1%—cells treated with 0.50 mg/mL concentrations of electrohydraulic-processed superparamagnetic iron oxide nanoparticles functionalized with doxorubicin and coated with citric acid. *p* < 0.005.

In this case (Figure 8a,b), two different concentrations of nanoparticles were compared. As expected, nanoparticles coated with citric acid and loaded with chemotherapy drugs are more effective. This pattern is also well expressed in time and as expected, the cytotoxic effect of the samples on RM1 line tumor cells is much higher at the concentration of 1.00 mg/mL.

The RM1 cell line exhibits increased resistance, as is stated in the literature [28], which elucidates why the concentrations of SPIONs that demonstrated efficacy in our previous studies may have yielded diminished cytotoxic effects in this context.

#### 3.2.2. Effects of Functionalized Nanoparticles on MEC1 Cell Lines

**Figure 9:**
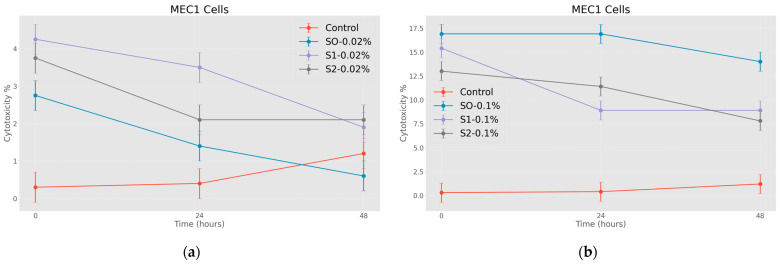
Effects of nanoparticles on MEC 1 cell line (MTT assay—cytotoxicity assessed at 570 nm), where control represents untreated tumor cells; (**a**) S0-0.02%—cells treated with 0.20 mg/mL concentrations of superparamagnetic iron oxide nanoparticles; S1-0.02%—cells treated with 0.20 mg/mL concentrations of superparamagnetic iron oxide nanoparticles coated with citric acid; S2-0.02%—cells treated with 0.20 mg/mL concentrations of electrohydraulic-processed superparamagnetic iron oxide nanoparticles coated with citric acid. (**b**) S0-0.1%—cells treated with 1.00 mg/mL concentrations of superparamagnetic iron oxide nanoparticles; S1-0.1%—cells treated with the 1.00 mg/mL concentrations of superparamagnetic iron oxide nanoparticles coated with citric acid; S2-0.1%—cells treated with—1.00 mg/mL concentrations of electrohydraulic-processed superparamagnetic iron oxide nanoparticles coated with citric acid. *p* < 0.005.

In the first stage of the research, the cytotoxic effect of nanoparticles coated with citric acid was determined. The obtained results emphasize the fact that the nanoparticles coated with citric acid have a certain cytotoxic effect on the MEC 1 cell line, and if they are added in the form where they are also loaded with the chemotherapy drug, it is assumed that this nanosystem will significantly increase the effectiveness of the test substance. The effect is also well timed and, in most cases, only fades slightly as time goes on. It is also worth noting the effectiveness of the drug coated with citric acid, which is detected immediately after treatment, but then the initial effect is overcome by tumor cells. The presented graph (Figure 9a,b) shows the cytotoxic effect of the nanosystems on tumor cells after the exposure at relatively high (1.00 mg/mL) and relatively low (0.20 mg/mL) concentrations.

**Figure 10:**
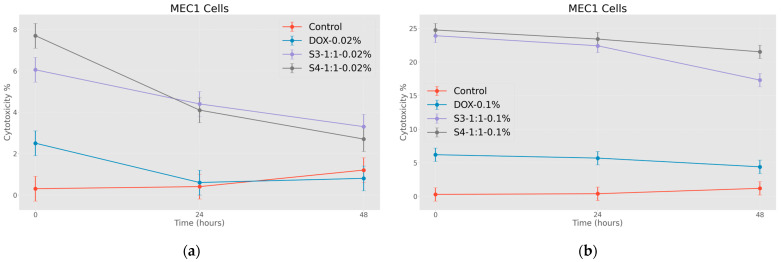
Effects of nanoparticles on MEC 1 cell line (MTT assay—optical density assessed at 570 nm), where control represents untreated tumor cells: (**a**) DOX-0.02%—cells treated with doxorubicin at 0.37 mg/mL concentration; S3-1:5-0.02%—cells treated with 0.17 mg/mL concentration of doxorubicin-functionalized citric-acid-coated superparamagnetic iron oxide nanoparticles; S4-1:5-0.02%—cells treated with 0.17 mg/mL concentration of electrohydraulic-processed superparamagnetic iron oxide nanoparticles coated with citric acid and loaded with doxorubicin (**b**) DOX-0.1%—cells treated with doxorubicin at 1.84 mg/mL concentration; S3-1:1-0.1%—cells treated with 0.83 mg/mL concentration of doxorubicin-functionalized citric-acid-coated superparamagnetic iron oxide nanoparticles; S4-1:1-0.1%—cells treated with 0.83 mg/mL concentration of electrohydraulic-processed superparamagnetic iron oxide nanoparticles coated with citric acid and loaded with doxorubicin. *p* < 0.005.

As for the cytotoxic effect of nanoparticles loaded with doxorubicin, two different concentrations were compared for conspicuous results. As expected, nanoparticles coated with citric acid are more effective after being loaded with chemotherapeutic drugs. The effect is also well expressed in time, and as expected, the cytotoxic effect of the nanosystems on tumor cells after the exposure is relatively high at the 1.00 mg/mL concentration (see Figure 10a,b).

Interesting data were obtained as a result of the research, emphasizing the effectiveness of nanoparticles. However, in order to conduct a complete study, it was desirable to evaluate the efficiency of nanosystems obtained in a different way. For this very reason, after confirming the cytotoxic efficiency of nanoparticles coated with citric acid, it became interesting to evaluate the effect of nanoparticles conjugated with folic acid. In particular, the nanosystems were loaded with doxorubicin and the cytotoxic effect on tumor cells was evaluated with the help of the same MTT immunofluorescence method. The cytotoxic effect of nanoparticles surrounded by folic acid and with a fewer number of modifications was determined. In this case, it is notable that we have obtained a slightly different picture.

**Figure 11:**
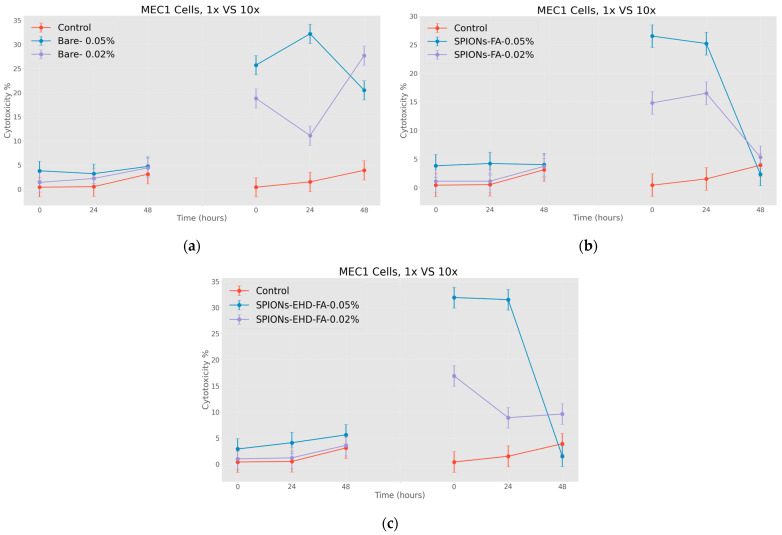
Effect of nanoparticles on tumor cells of MEC1 line, (MTT analysis—estimated optical density at 570 nm), where control represents untreated cells; (**a**) Bare—cells treated with solutions of 0.50 mg/mL and 0.20 mg/mL concentration of bare superparamagnetic iron oxide nanoparticles; (**b**) SPIONs-FA—cells treated with 1:10 concentration of 0.45 mg/mL and 0.18 mg/mL solutions of superparamagnetic iron oxide nanoparticles conjugated with folic acid; and, (**c**) SPIONs-EHD-FA—cells treated with 0.50 mg/mL and 0.20 mg/mL concentration of electrohydraulic-processed superparamagnetic iron oxide nanoparticles solutions conjugated with folic acid at 1:10 concentration. *p* < 0.005.

In the graphs below (Figure 11a–c), different effects are clearly expressed when nanoparticles are used in different concentrations. What is particularly noteworthy is the almost total disappearance of the antitumor effect of nanoparticles conjugated with folic acid after 48 h of incubation, where such an effect was not observed in the case of particles coated with citric acid.

**Figure 12:**
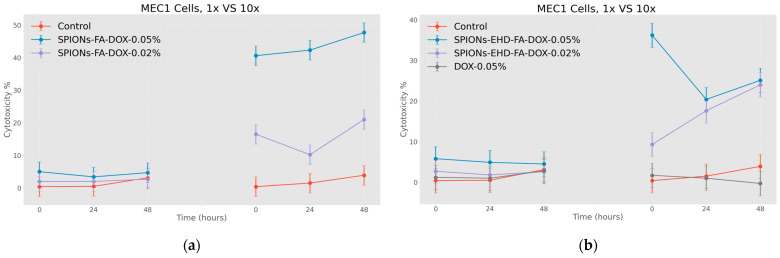
Effect of nanoparticles on tumor cells of MEC1 line (MTT analysis—estimated optical density at 570 nm), where control represents untreated cells; (**a**) SPIONs-FA-DOX—cells treated with 1:10 concentration of 0.45 mg/mL and 0.18 mg/mL solutions of superparamagnetic iron oxide nanoparticles loaded with doxorubicin and conjugated with folic acid; and (**b**) SPIONs-EHD-FA-DOX—cells treated with 1:10 concentration of 0.45 mg/mL and 0.18 mg/mL solutions of electrohydraulic-processed, folic-acid-conjugated and doxorubicin-loaded superparamagnetic iron oxide nanoparticles; DOX—cells treated with 1:10 concentration of 0.45 mg/mL solution of doxorubicin. *p* < 0.005.

It can be clearly seen that the use of the same concentration of doxorubicin that was loaded on the nanoparticles conjugated with folic acid (0.045 mg/mL) does not actually give a cytotoxic effect (Figure 12a,b), neither at low nor at high doses. This is a remarkable fact. Based on the results of these last studies, it was considered necessary to evaluate the ability of both doxorubicin (because it has the ability to fluoresce) and also the ability of nanoparticles loaded with doxorubicin to penetrate cells using fluorescence microscopy.

#### 3.2.3. Results of Fluorescence Microscopy Performed on MEC1 Cell Culture

In the next stage of the research, we evaluated how efficiently the doxorubicin-loaded nanoparticles reached the tumor cells. It was also of interest to see if there was any difference between the nanoparticles coated with citric acid and nanoparticles conjugated with folic acid.

**Figure 13:**
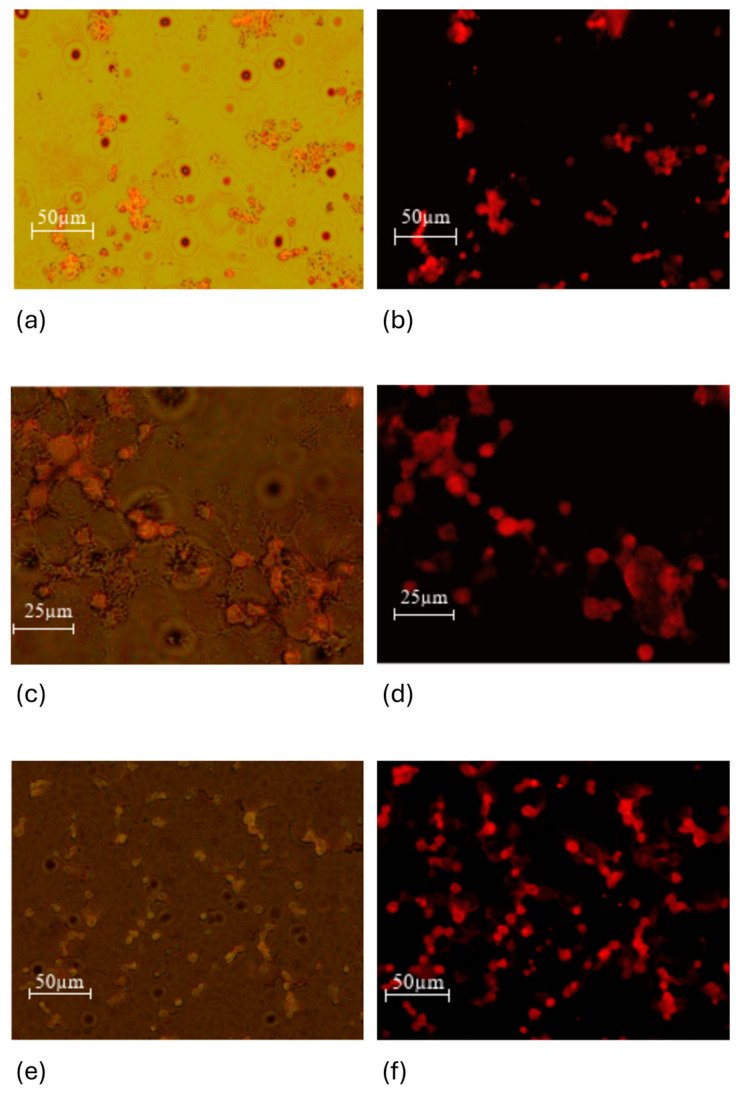
MEC1 cells were treated with different nanoparticles: (**a**) MEC1 cells and + DOX 0.05% concentration (0.045 mg/mL)—overlay; (**b**) MEC1 cells and + DOX 0.05% concentration (0.045 mg/mL)—green laser 532 nm; (**c**) MEC1 cells + SPIONs-FA-DOX-0.05% (0.045 mg/mL)—overlay; (**d**) MEC1 cells + SPIONs-FA-DOX-0.05% (0.045 mg/mL)—green laser 532 nm; (**e**) MEC1 cells + SPIONs-CA-DOX-0.05% (0.025 mg/mL)—overlay; and (**f**) MEC1 cells + SPIONs-CA-DOX-0.05% (0.025 mg/mL)—green laser 532 nm.

As can be seen from the microscopy photos (Figure 13a–f), when the drug enters the cells and the samples are irradiated with a green laser, doxorubicin fluoresces red, which makes it especially easy to detect the entry of the nanoparticles loaded with the chemotherapy drug into the cells. Although there is a necessity to dissociate doxorubicin from the SPIONs in order to facilitate cellular entry, our observations indicate that this dissociation does not lead to a decrease in the activity of doxorubicin on the samples.

## 4. Discussion

Our study demonstrated that citric-acid-coated nanoparticles, especially when loaded with doxorubicin, exhibit significant cytotoxic effects on both RM1 and MEC1 cell lines. This supports their potential as effective therapeutic agents for cancer treatment. Additionally, the enhanced cytotoxicity observed with doxorubicin-loaded nanoparticles validates their role in targeted drug delivery, minimizing off-target effects compared to conventional chemotherapy.

In our studies, we opted to utilize concentrations and modifications of SPIONs consistent with those employed in our previous research [11]. This methodological continuity allows for a reliable comparison of results, thereby enhancing the validity of our findings. It is well known that doxorubicin is a first-line anti-neoplastic agent that exhibits broad anticancer activity against various solid tumors as well as leukemias and lymphomas [31]. While it is effective in both types of cancers, it is particularly noted for its effectiveness against solid tumors such as breast, ovarian, bladder, and thyroid cancers. Its activity against leukemias is also significant, but the specific efficacy can vary based on the type of leukemia. The effectiveness of DOX increases significantly with time, highlighting the importance of treatment duration in achieving better therapeutic outcomes [32]. This explains the relatively low toxicity rates of the doxorubicin activity in this study compared to our previous one [11].

Our research highlighted a notable difference in efficacy between nanoparticles coated with citric acid and those conjugated with folic acid. Citric-acid-coated nanoparticles consistently maintained their cytotoxic effect over time of 48 h, whereas the effectiveness of folic-acid-conjugated nanoparticles diminished. This finding underscores the importance of coating selection in optimizing nanoparticle stability and effectiveness in therapeutic applications, as it is seen in the literature [33]. On the one hand, we have more stable citric-acid-coated nanoparticles, and on the other hand, targeted delivery with folic acid, which has also been recently discussed by other authors as well [34].

While the study demonstrates the promising potential of citric-acid-coated nanoparticles loaded with doxorubicin, it is important to acknowledge certain limitations. The in vitro studies, while valuable, do not fully capture the complex interactions that occur in vivo. Further research is needed to evaluate the biodistribution, pharmacokinetics, and long-term effects of these nanoparticles in animal models. Additionally, the study focused on two specific cancer cell lines. Expanding the investigation to include a wider range of cancer types would provide a more comprehensive understanding of the nanoparticles’ efficacy and potential for personalized medicine. Future research should also explore the potential for combining these nanoparticles with other therapeutic modalities, such as radiation therapy or immunotherapy, to enhance treatment outcomes.

The findings of this study highlight the potential of nanotechnology to revolutionize cancer treatment. The ability to target specific cancer cells with minimal off-target effects offers a significant advantage over conventional chemotherapy, which often leads to debilitating side effects. The use of nanoparticles for targeted drug delivery opens up exciting possibilities for personalized medicine, where treatment strategies can be tailored to the individual patient’s needs. Further research into the development of nanoparticle-based therapies could lead to more effective, less toxic, and more personalized cancer treatments, ultimately improving the lives of cancer patients.

## 5. Conclusions

This study demonstrates the promising potential of citric-acid-coated superparamagnetic iron oxide nanoparticles (CA-SPIONs) as effective therapeutic agents for cancer treatment, particularly when loaded with doxorubicin. The characterization results confirmed the stability and favorable properties of the nanoparticles, including appropriate zeta potential values and hydrodynamic diameters that suggest a well-dispersed system. Importantly, the cytotoxicity assessments revealed that CA-SPIONs significantly enhance the therapeutic efficacy against both RM1 and MEC1 cancer cell lines compared to non-coated and folic-acid-conjugated nanoparticles.

The results indicate a clear advantage of citric acid coatings over folic acid in maintaining therapeutic effects over time. While folic acid serves as a targeted delivery agent, its effectiveness appeared to diminish, highlighting the critical role that surface modifications play in nanoparticle stability and efficacy. Furthermore, the successful drug loading confirmed the potential of these nanoparticles to deliver chemotherapeutic agents directly to tumor sites, thereby minimizing the systemic toxicity that is often associated with conventional treatments.

Despite the positive findings, the study acknowledges limitations inherent to in vitro research and emphasizes the need for the further exploration of the long-term effects, biodistribution, and pharmacokinetics of CA-SPIONs and FA-SPIONs in vivo. Future research should also consider the incorporation of these nanoparticles into combination therapies, which may enhance treatment outcomes through synergistic effects.

In conclusion, our findings underscore the transformative potential of nanotechnology in oncology, paving the way for innovative, personalized approaches to cancer treatment. By leveraging the unique properties of nanoparticles for targeted drug delivery, we can envision a new paradigm in cancer therapy that enhances treatment efficacy while minimizing adverse effects, ultimately improving the quality of life for patients. Further investigation and development could lead to more effective, less toxic, and personalized cancer treatments, significantly impacting the future of oncology.

## Figures and Tables

**Table 1:** Designations of samples, their composition, and concentration.

Designations	Composition	Concentrations (mg/mL)
DOX-0.1%	Doxorubicin	1.84 mM
DOX-0.05%		0.92 mM
DOX-0.02%		0.37 mM
S0-0.1%	Bare nanoparticles	1.00 mg/mL
S0-0.05%		0.50 mg/mL
S0-0.02%		0.20 mg/mL
S1-0.1%	Citric-acid-coated nanoparticles	1.00 mg/mL
S1-0.05%		0.50 mg/mL
S1-0.02%		0.20 mg/mL
S2-0.1%	Electrohydraulically treated Citric-acid-coated nanoparticles	1.00 mg/mL
S2-0.05%		0.50 mg/mL
S2-0.02%		0.20 mg/mL
S3-1:1-0.1%	Citric-acid-coated nanoparticles functionalized with doxorubicin	0.50 mg/mL
S3-1:1-0.05%		0.25 mg/mL
S3-1:1-0.02%		0.10 mg/mL
S3-1:5-0.10%		0.83 mg/mL
S3-1:5-0.05%		0.42 mg/mL
S3-1:5-0.02%		0.17 mg/mL
S3-1:10-0.10%		0.91 mg/mL
S3-1:10-0.05%		0.45 mg/mL
S3-1:10-0.02%		0.18 mg/mL
S4-1:1-0.1%	Electrohydraulically treated Citric-acid-coated nanoparticles functionalized with doxorubicin	0.50 mg/mL
S4-1:1-0.05%		0.25 mg/mL
S4-1:1-0.02%		0.10 mg/mL
S4-1:5-0.1%		0.83 mg/mL
S4-1:5-0.05%		0.42 mg/mL
S4-1:5-0.02%		0.17 mg/mL
S4-1:10-0.1%		0.91 mg/mL
S4-1:10-0.05%		0.45 mg/mL
S4-1:10-0.02%		0.18 mg/mL
SPIONs-FA-0.05%	Folic-acid-conjugated nanoparticles	0.45 mg/mL
SPIONs-FA-0.02%		0.18 mg/mL
SPIONs-EHD-FA-0.05%	Electrohydraulically treated Folic-acid-conjugated nanoparticles	0.50 mg/mL
SPIONs-EHD-FA-0.02%		0.20 mg/mL
SPIONs-FA-Dox-0.05%	Folic-acid-conjugated nanoparticles functionalized with doxorubicin	0.45 mg/mL
SPIONs-FA-Dox-0.02%		0.18 mg/mL
SPIONs-EHD-FA-Dox-0.05%	Electrohydraulically treated Folic-acid-conjugated nanoparticles functionalized with doxorubicin	0.45 mg/mL
SPIONs-EHD-FA-Dox-0.02%		0.18 mg/mL

## Data Availability

The original contributions presented in the study are included in the article, further inquiries can be directed to the corresponding authors.
